# Injuries of the Portal Vein in Patients With Blunt Abdominal Trauma

**DOI:** 10.1155/1993/74027

**Published:** 1993

**Authors:** D. Henne-Bruns, B. Kremer, D. M. Lloyd, U. Meyer-Pannwitt

**Affiliations:** 1 Department of Surgery University of Kiel Arnold-Heller-Str. 7 Kiel 1 D-2300 Germany; 2 Department of Surgery University of Hamburg Germany

## Abstract

Between January 1987 and September 1991, 68 severely traumatized patients underwent emergency laparotomy because of blunt abdominal trauma. Intraoperatively, 54.4% of the patients had a major injury to one organ, 23.5% had injuries to two organs, 16.2% had injuries to three organs and 5.9% to four or more organs. Additionally, in 11.8% of these cases (*n* = 8) a major vascular injury (portal vein *n* = 5, vena cava *n* = 2, mesenteric root *n* = 1) was found. Injuries to the portal vein were always associated with complete rupture of the pancreas, requiring distal pancreatic resection in four cases and a duodenum preserving resection of the head of the pancreas in one. In two of these patients the portal vein had to be reconstructed with a Goretex prosthetic graft. Mortality was 14.7% for the whole group (*n* = 68) and 0% for patients with additional portal venous injuries.


*HPB Surgery*, 1993, Vol. 6, pp. 163–168
Reprints available directly from the publisher
Photocopying permitted by license only

© 1993 Harwood Academic Publishers GmbH
Printed in the United States of America

### INJURIES OF THE PORTAL VEIN IN PATIENTS WITH BLUNT ABDOMINAL TRAUMA

D. HENNE-BRUNS, B. KREMER, D.M. LLOYD* and U. MEYER-PANNWITT*

*Department of Surgery, University of Kiel and * Department of Surgery, University of Hamburg, Germany*

Between January 1987 and September 1991, 68 severely traumatized patients underwent emergency laparotomy because of blunt abdominal trauma. Intraoperatively, 54.4% of the patients had a major injury to one organ, 23.5% had injuries to two organs, 16.2% had injuries to three organs and 5.9% to four or more organs. Additionally, in 11.8% of these cases ($n$ = 8) a major vascular injury (portal vein $n$ = 5, vena cava $n$ = 2, mesenteric root $n$ = 1) was found. Injuries to the portal vein were always associated with complete rupture of the pancreas, requiring distal pancreatic resection in four cases and a duodenum preserving resection of the head of the pancreas in one. In two of these patients the portal vein had to be reconstructed with a Goretex prosthetic graft. Mortality was 14.7% for the whole group ($n$ = 68) and 0% for patients with additional portal venous injuries.

KEY WORDS: Multiple injuries, blunt abdominal trauma, portal vein injuries

### INTRODUCTION

In contrast to reports involving traumatic injuries of the thoracic aorta or iliac arteries[6], analyses of the incidence, localisation and management of complex abdominal venous injuries due to blunt trauma are still rare. Following the compulsory use of safety belts in Germany, the pattern of injuries in multiple traumatized patients seems to have changed from mainly head injuries to an increasing number of complex abdominal injuries. The injuries often include the simultaneous disruption of the liver, pancreas and spleen. These emergency situations require immediate exploration and a standardized concept for the operative procedure in order to achieve early hemostasis and optimal surgical intervention.

### PATIENTS AND OPERATIVE TECHNIQUES

Between January 1987 and September 1991, 68 patients with multiple trauma underwent emergency laparotomy due to severe abdominal injury at the Dept of Surgery, University of Hamburg. At laparotomy, 54.4% of the patients ($n$ = 37) had significant injury to one organ, 23.5% had injury to two organs ($n$ = 16), 16.2% to three organs ($n$ = 11) and 5.9% to four or more organs ($n$ = 4). The number of all injuries is listed in Table 1.

---

Address correspondence to: Prof. Dr Doris Henne-Bruns, Dept. of Surgery, University of Kiel, Arnold-Heller-Str. 7, D-2300 Kiel 1, FRG

**Table 1** Distribution of injured organs in 68 multiply traumatized patients.

| *Organ* | *No* |
|---|---|
| Spleen | $n$=7 |
| Liver/Gallbladder | $n$=34 |
| Perforation: | |
| Urinary bladder | $n$=3 |
| Small bowel, colon | $n$=16 |
| Pancreas | $n$=8 |
| Vascular injuries: | |
| Portal vein | $n$=5 |
| Vena Cava | $n$=3 |
| Iliac artery | $n$=2 |
| Mesenteric root | $n$=1 |
| Mesentery | $n$=7 |
| Diaphragm | $n$=10 |
| Kidney | $n$=2 |
| Ampulla of Vater | $n$=1 |

In 11.8% of the patients ($n$ = 8) additional vascular injuries were found involving the portal vein ($n$ = 5), the vena cava ($n$ = 2) and the mesenteric root ($n$ = 1). Injury to the portal vein in these five patients was always combined with a pancreatic rupture and liver trauma. Table 2 shows the pattern of injuries in these five patients who were admitted with severe hemorrhage and hemorrhagic shock. Three patients were involved in car accidents, all three being drivers who sustained steering wheel crush injuries. A fourth patient was a truck driver who was crushed between the truck and the trailer of the truck; the fifth patient was a truck driver involved in a head-on collision who was impailed in the abdomen by steel girders.

After attempting immediate resuscitation, the patients had emergency ultrasound investigation and / or diagnostic lavage to confirm intraabdominal bleeding before proceeding to laparotomy. In all five patients a temporary stabilization of their cardiovascular status was achieved, and a transverse incision with extension into an upper mid-line incision was performed. The exploration of the abdominal cavity began with the systematic packing of all four quadrants starting first around the spleen, then the liver, upper abdomen and lower abdomen, respectively. In all five patients liver injuries also occurred. The falciform ligament was rapidly divided in order to facilitate the packing. When hemostasis was controlled, the exploration and operation began at the spleen and / or the pancreas. Because all 5 patients were suspected of having complex upper abdominal injuries, possibly involving the portal vein and its tributaries, the exploration began with inspection of the spleen and pancreas after division of the greater omentum. Only when injuries to these organs and their veins were repaired was crossclamping of the portal structures performed.

Splenectomy and distal pancreatic resection was necessary in patients 1,3,4 and 5. The extent of pancreatic resection was determined by the site of the injury which, in all 5 cases, occurred at the junction of the neck and body overlying the vertebral column. In patients 1 and 4 a Goretex prosthesis had to be grafted between the superior mesenteric vein and the portal vein following the pancreatic resection, because the extensive defect could not be reconstructed despite full

**Table 2** Survey of injured organs and operative procedures in patients with abdominal trauma involving vascular (venous) injuries.

| *Patient:* | *Trauma* | *Operation* |
|---|---|---|
| 1 | Rupture of portal vein, pancreas, liver, spleen | Resection of left lobe of the liver, left pancreatectomy, splenectomy, reconstruction of the portal vein and prosthetic graft implantation |
| 2 | Rupture of portal vein, liver, duodenum, pancreas, ampulla of vater | Duodenum preserving resection of the head of the pancreas, cholecystectomy, suturing of the liver parenchyma, oversewing of the portal vein |
| 3 | Rupture of portal, vein, liver, pancreas | Distal pancreatectomy, splenectomy, liver suturing, reanastomosis of the portal vein |
| 4 | Rupture of portal vein, liver, pancreas, colon | Distal pancreatectomy, splenectomy, liver suturing, colon suturing, reconstruction of the portal vein and prosthetic graft implantation |
| 5 | Rupture of portal vein, liver, pancreas, spleen | Distal pancreatectomy, splenectomy, suturing of the liver, oversewing of the portal vein |

mobilization (Figure 1). In patient 3, a direct anastomosis between the portal vein and the superior mesenteric vein was possible; in patient 5 the portal vein was oversewn following complete avulsion of the splenic vein.

The fifth patient in this group (patient no. 2), had complex injuries to the duodenum, head of the pancreas as well as disruption of the portal vein. In addition, the ampulla of Vater was avulsed from the duodenum. In this case a duodenum preserving resection of the head of the pancreas was performed. The portal vein was oversewn, the distal bile duct was resected and the proximal duct anastomosed to a Roux-en-Y loop together with the distal pancreas.

After completion of the pancreatic resections and repair of the vascular structures the packs from around the liver were removed. In order to evaluate the extent of the hepatic injury as well as to explore the retrohepatic caval vein and the hepatic veins, the liver was mobilized by division of the coronary and triangular ligaments. The hepato-duodenal ligament was crossclamped and the injuries were repaired either by suturing of the parenchyma (4 patients 2 to 5 lacerations in the right lobe 1–2 cm deep) or by liver resection (1 patient, left lateral liver resection).

### RESULTS

Mortality for all patients with blunt abdominal trauma ($n$ = 68) was 14(7%), and for patients with additional portal vein injury 0% ($n$ = 5). The mean number of intraoperative blood transfusions was 16 (12–23) units.

**Figure 1** Photograph demonstrating Goretex prosthesis between the superior mesenteric vein (SMV) and portal vein (PV) following distal pancreatectomy. SV = Splenic Vein (stump), IMV = inferior mesenteric vein (stump). *(See colour plate at the back of this issue)*

The morbidity in patients who sustained portal venous injuries includes 1 case (patient 2) with a biliary fistula, which healed spontaneously after 8 weeks. One patient who had a reconstruction with a Goretex prosthesis underwent secondary closure of the abdomen 48 hours after the initial procedure. The prolonged crossclamping of the superior mesenteric vein, induced congestion and dilatation of the small bowel, prohibiting primary closure. In 3 patients the postoperative course was completely uneventful. Duplex sonography in both patients in which the portal vein was reconstructed with the synthetic graft demonstrated normal portal venous flow three months after surgery.

### DISCUSSION

Traumatic injuries to the portal vein are rarely reported in the European literature. Lauterjung[6] did not report any venous injuries in a series of 296 patients with blunt abdominal trauma. Farthmann[4] described 17 vascular injuries (7 × artery, 10 × vein) in 14 patients who sustained multiple trauma in a series of 291 patients. In both series the abdominal trauma was due to blunt forces in 96% and 99(4%), respectively. Penetrating injuries were only observed in 4% and 0.6% respectively.

In the North American literature, both portal vein and vena cava injuries are reported frequently. 80–90% of these injuries are due to penetrating (gun shot or stab wound) trauma[1,2,7,8,10,12].

Because complex blunt abdominal trauma involving vascular lesions is rarely observed, and because the pattern of injured organs is extremly variable, a standardized therapeutic procedure cannot be constructed to suit all cases. However, conceptually, all 4 abdominal quadrants should be packed to achieve an initial hemostasis and allow systematic exploration[10]. In combined injuries of the spleen, pancreas, portal vein and liver the repair should begin in the left upper quadrant. If this is not done, crossclamping the hilum of the liver would induce severe hemorrhage from the spleen, pancreas or portal vein because of acute venous congestion. For this reason we strongly disagree with the use of portal vein ligation in complex portal venous injuries as reported by some authors[4,12].

For reconstruction of the portal vein either direct anastomosis, lateral venorrhaphy or interposition with a graft should be considered[9]. In cases where a direct anastomosis or lateral venorrhaphy can be performed we would prefer this technique, because a fast reconstruction can be achieved. Graft interposition can be performed either by autologous or synthetic material. Because it is difficult to isolate an autologous graft of adequate diameter for reconstruction of the portal vein in an emergency situation, we would consider the use of a synthetic graft interposition as faster and perhaps more beneficial in these circumstances. Because the superior mesenteric vein has to be crossclamped during the reconstruction period the prolonged extent of venous congestion of the small bowel might increase postoperative complications.

Following the resection of the pancreas and the reconstruction of the adjacent vessels the liver and the retrohepatic vena cava have to be explored. Therefore, the falciform ligament and the coronary and triangular ligaments have to be divided. Because these structures are usually not very well vascularized their division can be performed expeditiously. The right lobe can be mobilized to allow complete evaluation of the retrohepatic vena cava. In contrast to several reports in the literature[4,12] a rapid exposure of the retrohepatic vena cava was possible in all cases. A thoraco-abdominal approach, as described by Coln[3] and Schrock[11], is not necessary, because complete mobilization of both liver lobes allows excellent access to the cava, which can be isolated or crossclamped if necessary. Only in injuries which involve the intrathoracic caval vein, is a purely abdominal approach insufficient. Unfortunately these injuries are so severe that death usually prevails, because of the absence of tamponade. In our series the complete mobilisation and exploration of the liver facilitated the final treatment, which consisted of one left lateral resection and suturing of the right lobe parenchyma in 4 cases.

Mortality of abdominal trauma involving portal venous injuries is reported to be up to 70%[7,9,10,12]. It is difficult to compare most series because of the small numbers of patients sustaining incomparable complex venous injuries caused by a great variety of trauma. Most of the cases with portal vein injury are complex lesions caused by stab or gunshot wounds near the porta hepatis[2,5,9]. The combined injuries of the portal vein, bile duct and hepatic artery cannot be directly compared to the pattern of injuries caused by blunt abdominal trauma which often involves the pancreas. In our limited series the mortality was surprisingly 0%. None of the patients developed severe pancreatitis after the blunt pancreatic trauma and pancreatic resection.

A rapid appraisal of complex abdominal injuries with possibly a reduction in the mortality rate can be attained if primary hemostasis with packing is achieved. This allows time for a definite surgical strategy to be established. Our series demonstrates that this systematic approach in severely injured patients leads to an acceptable mortality and morbidity. We would recommend its use for all patients with severe abdominal trauma, where complex venous injuries are suspected.

### *References*

1. Agarwal, N., Shah, P.M., Clauss, R.H., Reynolds, B.M., Stahl, W.M. (1982) Experience with 115 Civilian Venous Injuries. *J. Trauma*, **22** (10), 827–831
2. Busuttil, R.W., Kitahama, A., Cerise, E., McFadden, M., Lo, R. and Longmire, W.P. (1980) Management of blunt and penetrating injuries to the porta hepatis. *Ann. Surg.*, **191**, 641–648
3. Coln, D., Crighton, J. and Schorn, L. (1980) Successful Management of Hepatic Vein Injury From Blunt Trauma in Children. *Am. J. Surg.*, **140**, 858–864
4. Farthmann, E.H., Kirchner, R. and Fraedrich, G. (1989) Organ-und Gefässverletzungen des zentralen Retroperitoneums. *Chirurg.*, **60**, 657–664
5. Ivatury, R.R., Nallathambi, M., Lankin, D.H., Wapnir, I., Rohman, M. and Stahl, W.M. (1987) Portal vein injuries. *Ann. Surg.*, **206**, 733–737
6. Lauterjung, K.L., Hofmann, G.O., Mittlmeier, Th. and Huf, R. (1987) Thorax- und Abdominalverletzungen beim Polytrauma. *Chirurg.*, **58**, 641–647
7. Mattox, K.L., Espada, R. and Beall, A.C. (1975) Traumatic Injury to the Portal Vein. *Ann. Surg.*, **181** (5), 519–521
8. Millikan, J.S., Moore, E.E., Cogbill, T.H. and Kashuk, J.L. (1983) Inferior Vena Cava Injuries - A Continuing Challenge. *J. Trauma*, **23** (3), 207–211
9. Pachter, H.L., Drager, S., Godfrey, N. and LeFleur, R. (1979) Traumatic injuries of the portal vein. *Ann. Surg.*, **189**, 383–385
10. Petersen, S.R., Sheldon, G.F. and Lim, R.C. (1979) Management of Portal Vein Injuries. *J. Trauma*, **19** (8), 616–619
11. Schrock, T., Blaisdell, F.W. and Mathewson, C. (1968) Management of Blunt Trauma to the Liver and Hepatic Veins. *Arch. Surg.*, **96**, 698–704
12. Stone, H.H., Fabian, T.C. and Turkleson, M.L. (1982) Wounds of the Portal Venous System. *World J. Surg.*, **6**, 335–341

*(Accepted by S. Bengmark 1 August 1992)*

